# Headspace, Volatile and Semi-Volatile Organic Compounds Diversity and Radical Scavenging Activity of Ultrasonic Solvent Extracts from *Amorpha fruticosa* Honey Samples

**DOI:** 10.3390/molecules14082717

**Published:** 2009-07-27

**Authors:** Igor Jerković, Zvonimir Marijanović, Janja Kezić, Mirko Gugić

**Affiliations:** 1 Faculty of Chemistry and Technology, University of Split, N. Tesle 10/V, 21000 Split, Croatia; 2 Marko Marulić Polytechnic in Knin, P. Krešimira IV 30, 22300 Knin, Croatia E-mails: zmarijanovic@veleknin.hr (Z.M.); jkezic@veleknin.hr (J.K.); mgugic@veleknin.hr (M.G.)

**Keywords:** *Amorpha fruticosa* honey volatiles, headspace solid-phase microextraction (HS-SPME), ultrasonic solvent extraction (USE), gas chromatography and mass spectrometry (GC, GC-MS), DPPH scavenging activity

## Abstract

Volatile organic compounds of *Amorpha fruticosa* honey samples were isolated by headspace solid-phase microextraction (HS-SPME) and ultrasonic solvent extraction (USE), followed by gas chromatography and mass spectrometry analyses (GC, GC-MS), in order to obtain complementary data for overall characterization of the honey aroma. The headspace of the honey was dominated by 2-phenylethanol (38.3–58.4%), while other major compounds were *trans*- and *cis*-linalool oxides, benzaldehyde and benzyl alcohol. 2‑Phenylethanol (10.5–16.8%) and methyl syringate (5.8–8.2%) were the major compounds of ultrasonic solvent extracts, with an array of small percentages of linalool, benzene and benzoic acid derivatives, aliphatic hydrocarbons and alcohols, furan derivatives and others. The scavenging ability of the series of concentrations of the honey ultrasonic solvent extracts and the corresponding honey samples was tested by a DPPH (1,1-diphenyl-2-picrylhydrazyl) assay. Approximately 25 times lower concentration ranges (up to 2 g/L) of the extracts exhibited significantly higher free radical scavenging potential with respect to the honey samples.

## Introduction

Unifloral honeys differ from each other, among other features, in volatile compound composition, which influences remarkably the individual sensory characteristics of each honey type. Consequently, the volatile organic compounds (VOCs) exhibit a potential role in distinguishing honeys as a function of botanical origin. In this regard, certain naturally occurring VOCs are characteristic of the botanical origin and can be considered as specific markers, such as methyl anthranilate for citrus honey [1], 2-hydroxy-5-methylhexan-3-one and 3-hydroxy-5-methylhexan-2-one for eucalyptus honey [2] or 2-aminoacetophenone for chestnut honey [3]. In other cases [4,5,6], the honey floral origin was characterized by a greater concentration or the absence of certain compounds (terpenes, norisoprenoids, benzene derived compounds and others). More than 600 organic compounds have been identified in different honey volatile flavors originated from various biosynthetic pathways. Aldehydes, ketones, esters, alcohols, hydrocarbons, and sulfur compounds are the common groups of volatiles that have been detected in all honeys. Among them, (*E*)-β-damascenone, phenylacetaldehyde, 2-phenylethanol linalool, *p*-anisaldehyde and benzaldehyde were reported to be important contributors to honey flavor [7,8]. Appropriate methods for honey VOC isolation should be applied since artifacts can be generated due to the effects of heat on honey carbohydrates and amino acids (Strecker degradation and Maillard or non-enzymatic browning reactions). In addition, there is a varying degree of selectivity and effectiveness toward honey VOCs isolated by different methods, depending on the compounds involved and the extraction conditions [9]. 

Honey serves as a source of natural antioxidants [10]. In the DPPH (1,1-diphenyl-2-picrylhydrazyl) radical system an antioxidant directly reacts with the radical, so the DPPH method has been widely used for determination of radical scavenging ability, which is one of the well known beneficial effects of honey, albeit greatly influenced by its botanical origin [11,12]. The honey radical scavenging ability has been strongly correlated with the content of total phenolics [13,14]. The components in honey responsible for its antioxidative effect are flavonoids, phenolic acids, ascorbic acid, catalase, peroxidase, carotenoids, amino acids, Maillard reaction products and others. To the best of our knowledge, there are no reports about the possible radical scavenging effects of any honey ultrasonic solvent extracts.

*Amorpha fruticosa* unifloral honey samples were selected for the present research according to their pollen analysis. The VOC diversity of this honey has not yet been studied. Based on our previous papers [15,16], headspace solid-phase microextraction (HS-SPME) and ultrasonic solvent extraction (USE) were applied as appropriate comprehensive methods for isolation of VOCs from the honey matrix. Isolated VOCs were analyzed by gas chromatography and mass spectrometry (GC and GC-MS) to obtain VOCs chemical fingerprints of the honey samples. In addition, the obtained USE extracts of each honey sample were tested by DPPH radical scavenging assay for the first time in order to unlock their radical scavenging potential for further more detail research. DPPH radical scavenging activity of the USE extracts were compared to the activity of the honey samples in order to evaluate its possible extra value (not just for analytical purposes) in comparison to the honey.

## Results and Discussion

The botanical origin of all *A. fruticosa* honey samples was determined by pollen analysis according to EU regulations [17,18]. The samples contained the pollen of *A. fruticosa* (47–53%), *Acer* spp. (4–11%), *Brassica napus* var. *oleifera* (0–10%), *Robinia pseudoacacia* (6–10%), *Trifolium pratense* (0–8%), *Trifolium repens* (0–7%), Asteraceae (0–7%), *Plantago* spp. (0–4%), Poaceae (0–2%), *Cornus sanguinea* (0-9%) and Apiaceae (0-3%). Electric conductivity of the samples was in the range 0.20–0.24 S.cm^-1^ and water content was in the range 16.4–17.4%.

HS-SPME and USE methods applied for VOC extraction from the honey matrix exhibited a varying degree of selectivity and effectiveness, depending upon the compounds involved and extraction conditions. Both methods operated at room temperature up to 40 ^o^C and enabled comprehensive isolation of more and less volatile compounds without generating thermal artifacts, that is crucial for obtaining reliable honey VOCs chemical compositions. 

### Diversity of volatiles isolated by headspace solid-phase microextraction (HS-SPME)

**Figure 1:**
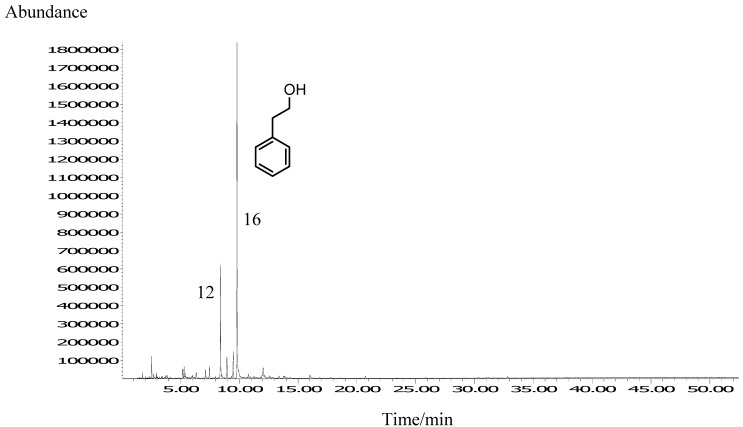
Representative TIC chromatogram of *A. fruticosa* honey headspace VOCs isolated by HS-SPME. Numbers refer to the compounds listed in Table 1.

A total of 25 VOCs were identified in *A. fruticosa* honey headspeace by HS-SPME followed by GC and GC-MS. A representative chromatogram is presented in Figure 1. 

Benzene derivatives were the most abundant compounds in the headspace of *A. fruticosa* honey, including 2-phenyethanol (38.3–58.4%), benzaldehyde (1.3–4.9%), phenylacetaldehyde (1.7–2.2%) and benzyl alcohol (1.1–1.3%), which have also been reported as ubiquitous in most honeys from a wide range of floral sources. However, very high percentage of 2-phenylethanol can be noted as characteristic of this honey, although it is not specific marker. This aromatic compound, with a floral odor, was identified as a potent contributor to the aroma of linden, haze, caju, morrăo de candeia and assa-peixe honeys [7,19,20]. Terpenes, particularly linalool derivatives, were another abundant group of the headspace VOCs (Table 1) including *trans*-linalool oxide (10.8–16.9%), *cis*-linalool oxide (1.3–3.2%), hotrienol (4.3–5.3%) and an isomer of lilac aldehyde (0.3–1.1%). 

**Table 1:** *A. fruticosa* honey VOCs isolated by HS-SPME.

No.	Compound	RI	Area percentage (%)			
			Min.	Max.	Av.	SD.
1.	Methyl sulfide	‹ 900	0.0	0.3	0.13	0.15
2.	Dimethyl disulfide	‹ 900	0.0	0.2	0.10	0.10
3.	Octane^a^	‹ 900	0.9	1.6	1.33	0.38
4.	Furfural^a^	‹ 900	0.5	1.4	0.97	0.45
5.	Nonane^a^	900	0.0	0.5	0.27	0.25
6.	1-(2-Furanyl)- ethanone	914	0.0	0.2	0.10	0.10
7.	Benzaldehyde^a^	965	1.3	4.9	2.97	1.81
8.	2,4-Dimethyl-3,6-dihydro-2H-pyran	969	1.0	1.6	1.20	0.35
9.	Hexanoic acid^a^	974	0.4	0.7	0.50	0.17
10.	Benzyl alcohol^a^	1037	1.1	1.3	1.20	0.10
11.	Phenylacetaldehyde^a^	1048	1.7	2.2	2.00	0.26
12.	*trans*-Linalool oxide (furan type)	1076	10.8	16.9	14.23	3.12
13.	*cis*-Linalool oxide (furan type)	1091	1.3	3.2	2.37	0.97
14.	Linalool^a^	1101	1.0	2.1	1.53	0.55
15.	Hotrienol	1106	4.3	5.3	4.80	0.50
16.	2-Phenylethanol^a^	1116	38.3	58.4	47.30	10.21
17.	Isophorone^a^	1124	0.1	1.2	0.67	0.61
18.	Phenylacetonitrile	1142	0.8	1.0	0.90	0.10
19.	4-Ketoisophorone	1147	0.0	0.4	0.17	0.21
20.	Lilac aldehyde^**^	1154	0.3	1.1	0.73	0.40
21.	Pinocarvone	1166	0.1	0.9	0.57	0.42
22.	Nonan-1-ol^a^	1175	1.0	2.8	1.77	0.93
23.	Nonanoic acid^a^	1273	0.7	2.1	1.43	0.70
24.	4-Vinyl-2-methoxy-phenol	1314	0.0	0.6	0.30	0.30
25.	(*E*)-β-Damascenone	1385	0.7	1.5	1.07	0.40
	**Total identified**		89.7-92.9%			

RI = retention indices on HP-5MS column; Min. = minimal percentage; Max. = maximal percentage; Av. = average percentage; SD. = standard deviation; ^a^ –identification confirmed with reference compound; ^**^ - correct isomer not identified.

Biogenetic studies have shown that lilac aldehydes are formed from linalool by direct hydroxylation of linalool at C8 to (*E*)-8-hydroxylinalool and further to (*E*)-8-oxolinalool, that is converted to lilac alcohols that undergo oxidation to lilac aldehydes [21]. Alternatively, epoxidation of linalool gives 6,7-epoxylinalool which undergoes further oxidation and heat and/or acidic conditions form two furan linalool oxides [22]. Minor percentages (4.3–5.3%) of hotrienol were also identified. Since no heat was applied during the VOC isolation process, hotrienol was probably formed during honey ripening [23]. Consequently, the percentages of furan derivatives, indicators of heat treatment, were low, with the major compound being furfural (0.5–1.5%). Minor percentages of norisoprenoids were found: isophoropne (0.1–1.2%), 4-ketoisophorone (0.0–0.4%) and (*E*)-β-damascenone (0.7–1.5%).

### Diversity of volatiles isolated by ultrasonic solvent extraction (USE)

**Figure 2:**
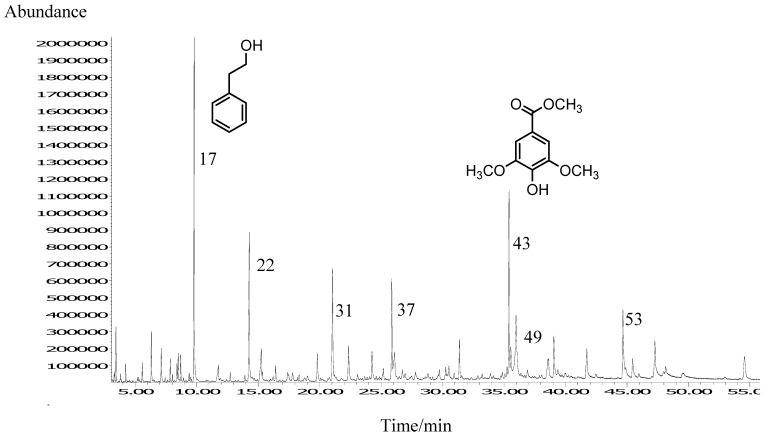
Representative TIC chromatogram of *A. fruticosa* honey VOCs isolated by USE. Numbers refer to the compounds listed in Table 2.

A total of 57 volatile and semi-volatile compounds were identified in *A. fruticosa* honey isolated by USE followed by GC and GC-MS. A representative chromatogram is presented in Figure 2. 

2-Phenylethanol was the major compound of USE extracts from all *A. fruticosa* honey samples, with percentages ranging from 12.1 to 16.8%, followed by minor percentages of other benzene derivatives such as benzyl alcohol (0.5–1.3%) and phenylacetaldehyde (0.0–0.1%). Another abundant compound was 2-phenylacetamide (3.2–7.4%), that is described as one of the major odor compounds of honey, irrespective of its floral origin [24]. More important compounds (although not so abundant) with respect to the honey botanical origin were shikimate pathway derivatives such as methyl syringate (5.8–8.2%), benzoic acid (0.5–1.1%), phenylacetic acid (0.8–2.1%), 4-hydroxybenzoic acid (0.9–2.3%), 4-hydroxyphenylacetic acid (0.2–0.7%), ferulic acid (0.6–1.2%) and others (Table 2). Methyl syringate was detected as the second most abundant compound of *A. fruticosa* honey volatiles and was present in the honeys obtained from plants of different botanical families [25], but only in asphodel honey did it reach the highest level [26]. However, in comparison with other unifloral honeys neither of the previous compounds can be emphasized as specific marker of *A. fruticosa* honey. 

**Table 2:** *A. fruticosa* honey VOCs isolated by USE.

No.	Compound	RI	Area percentage (%)			
			Min.	Max.	Av.	SD.
1.	4-Methyloctane^*^	‹ 900	0.1	0.2	0.13	0.06
2.	Ethylbenzene	‹ 900	0.3	0.4	0.33	0.06
3.	1,4-Dimethylbenzene (*p*-Xylene)	‹ 900	1.3	2.0	1.60	0.36
4.	Ethenylbenzene	‹ 900	0.0	0.2	0.10	0.10
5.	1,3-Dimethylbenzene (*m*-Xylene)	‹ 900	0.2	0.5	0.33	0.15
6.	Methoxybenzene^a^ (Anisol)	920	0.3	0.5	0.37	0.11
7.	Benzaldehyde^a^	965	0.0	0.1	0.07	0.06
8.	2,3-Dihydro-3,5-dihydroxy-6-methyl-4H-pyran-4-one	980	0.4	0.6	0.50	0.10
9.	2-Ethylfuran	982	0.7	2.3	1.57	0.70
10.	Benzyl alcohol^a^	1037	0.5	1.3	0.80	0.44
11.	Phenylacetaldehyde^a^	1048	0.0	0.1	0.07	0.06
12.	*trans*-Linalool oxide (furan type)	1076	0.3	0.7	0.53	0.21
13.	4,5-Dimethyl-2-formylfuran	1078	0.2	0.9	0.47	0.38
14.	1-(2-Furanyl)-2-hydroxyethanone	1084	0.4	1.0	0.63	0.32
15.	*cis*-Linalool oxide (furan type)	1091	0.0	0.1	0.06	0.05
16.	Nonanal^a^	1105	0.0	0.2	0.10	0.10
17.	2-Phenylethanol^a^	1116	10.5	16.8	13.47	3.17
18.	Benzoic acid^a^	1162	0.5	1.1	0.73	0.32
19.	4-Methylbenzyl alcohol^a^	1171	0.0	0.2	0.10	0.10
20.	3,7-Dimethylocta-1,5-dien-3,7-diol	1191	0.0	0.4	0.20	0.20
21.	4-Vinylphenol^a^	1221	0.2	0.3	0.27	0.06
22.	5-Hydroxymethylfurfural^a^	1230	3.5	9.1	6.53	2.83
23.	Phenylacetic acid^a^	1269	0.8	2.1	1.33	0.68
24.	1,4-di-*tert*-Butylbenzene	1257	0.0	0.4	0.20	0.20
25.	Nonanoic acid^a^	1273	0.0	0.1	0.06	0.05
26.	Benzene-1,4-diol	1278	0.0	0.4	0.20	0.20
27.	1-Phenylethan-1,2-diol	1304	0.2	0.7	0.50	0.26
28.	4-Vinyl-2-methoxyphenol	1314	0.0	0.6	0.37	0.32
29.	1-Hydroxylinalool	1365	0.0	1.8	1.03	0.93
30.	Butoxyethoxyethyl acetate	1371	0.0	0.2	0.07	0.11
31.	2-Phenylacetamide	1393	3.2	7.4	5.50	2.13
32.	4-Hydroxy-3-methoxybenzaldehyde^a^ (Vanilline)	1397	0.0	0.2	0.10	0.10
33.	4-Hydroxybenzyl alcohol^a^	1426	0.8	2.0	1.40	0.60
34.	4-Methoxybenzaldehyde^a^	1445	0.2	0.4	0.30	0.10
35.	Dodecan-1-ol^a^	1479	0.0	1.3	0.53	0.68
36.	Pentadecane^a^	1500	0.1	0.8	0.40	0.36
37.	4-Methyl-2,6-bis(1,1-dimethylethyl)-phenol	1514	2.9	4.2	3.60	0.66
38.	4-Hydroxybenzoic acid^a^	1522	0.9	2.3	1.47	0.73
	(*p*-Salicylic acid)					
39.	4-Hydroxyphenylacetic acid^a^	1563	0.2	0.7	0.47	0.25
40.	4-Hydroxy-3-methoxybenzoic acid^a^ (Vanillic acid)	1566	0.2	0.6	0.43	0.21
41.	4-Hydroxy-3,5-dimethoxy-benzaldehyde (Syringyl aldehyde)	1661	0.0	1.7	0.73	0.87
42.	Tetradecan-1-ol^a^	1678	0.0	0.4	0.13	0.23
43.	Methyl 3,5-dimethoxy-4-hydroxybenzoate^a^ (Methyl syringate)	1774	5.8	8.2	7.40	1.39
44.	3-(4-Hydroxyphenyl)-prop-2-enoic acid (4-Hydroxycinnamic acid)	1789	0.0	0.6	0.20	0.35
45.	4-Hydroxy-3,5,6-trimethyl-4-(3-oxo-1-butenyl)-cyclohex-2-en-1-one	1790	1.4	7.5	3.17	3.77
46.	3,5-Dimethoxy-4-hydroxybenzoic acid (Syringic acid)	1817	0.0	0.6	0.23	0.32
47.	3-(4-Hydroxy-3-methoxyphenyl)- prop-2-enoic acid^a^ (Ferulic acid)	1867	0.6	1.2	0.83	0.32
48.	Diisobutyl phthalate	1869	0.6	1.0	0.77	0.21
49.	Hexadecan-1-ol^a^	1882	2.5	8.2	5.23	2.86
50.	2,5-Dimethoxy-4-ethylbenzaldehyde	1890	0.0	0.4	0.13	0.23
51.	3-Hydroxy-4-methoxycinnamic acid (Isoferulic acid)	1869	0.0	0.6	0.27	0.31
52.	Hexadecanoic acid^a^	1963	1.4	1.9	1.70	0.26
53.	(*Z*)-Octedec-9-en-1-ol^a^	2060	5.2	16.8	11.87	5.99
54.	Octadecan-1-ol^a^	2084	1.4	2.6	2.00	0.60
55.	Heneicosane^a^	2100	0.0	3.9	1.97	1.95
56.	(*Z*)-Octadec-9-enoic acid^a^	2147	0.0	2.5	1.50	1.33
57.	Tetracosane^a^	2400	1.0	2.0	1.67	0.58
	**Total identified**		90.7-96.3%			

RI = retention indices on HP-5MS column; Min. = minimal percentage;Max. = maximal percentage; Av. = average percentage; SD. = standard deviation; ^a^ – identification confirmed with reference compound; ^*^ - tentatively identified; ^**^ - correct isomer not identified.

Higher aliphatic alcohols and acids were the second most abundant class of VOCs of the USE extracts, particularly hexadecan-1-ol (2.5–6.6%), (*Z*)-octadec-9-en-1-ol (5.2–16.8%) and octadecan-1-ol (1.4–3.5%). Their presence was expected, due probably to their possible beeswax origin and they cannot be considered important for botanical origin determination, as indicated in a previous paper [16]. A low percentage of furan derivatives were identified, including 5-hydroxymethylfurfural (3.5–9.1%), 2-ethylfuran (0.7–2.3%) and 4,5-dimethyl-2-formylfuran (0.2–0.9%), not as the honey markers but rather as indicators of heat treatment and prolonged or improper storage conditions. 

### Free radical scavenging ability of ultrasonic solvent extracts compared to the honey samples

**Figure 3:**
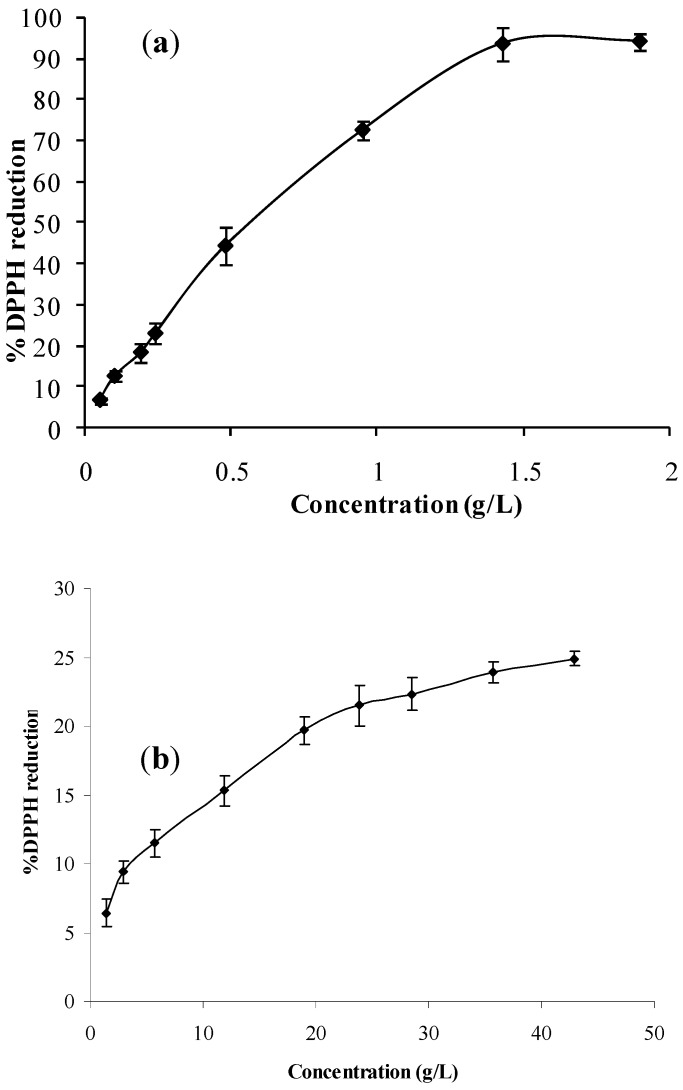
DPPH reduction percentage against increasing concentration of *A. fruticosa* USE extracts (**a**) and the honey samples (**b**) Results are expressed as mean values ± SD.

The 1,1-diphenyl-2-picrylhydrazyl (DPPH) assay was used to evaluate the free radical scavenging ability of the ultrasonic solvent extracts, as well as the honey samples. The decrease in absorbance was measured at room temperature after 1 h. The percent inhibition of the DPPH radical as a function of the honey samples and their ultrasonic solvent extracts concentrations is shown in Figure 3. The term IC_50_ corresponds to the concentration of the extract where 50% of its maximal effect on reduction of DPPH radical is observed. IC50 value was determined graphically from the graph in Figure 3(a). The IC_50_ value for ultrasonic solvent extract mean curve [Figure 3(a)] was 0.6 g/L, while the IC_50_ for the honey samples was undetermined because at the maximum concentration (45 g/L) a maximum of 25% of DPPH inhibition was achieved. These measurements show that the ultrasonic solvent extracts exhibited significantly higher free radical scavenging potential than the honey samples at approximately 25 times lower concentration ranges (up to 2 g/L). 

## Experimental

### Honey samples

In total of four honey samples were investigated. The samples were obtained from professional beekeepers and no mechanical treatment or heat was used. Melissopalynological analysis was performed by the methods recommended by the International Commission for Bee Botany [18]. Microscopical examination was carried out on a Hund h 500 (Wetzlar, Germany) light microscope attached to a digital camera (Motic m 1000) and coupled to an image analysis system (Motic Images Plus software) for morphometry of pollen grains. Each sample was examined to determine the percentage of pollen grains. Water content was determined by refractometry, measuring the refractive index, using a standard model Abeé refractometer at 20 ºC. Water content (%) was obtained from the Chataway table [18]. Electrical conductivity was measured in a solution of 20 g honey in low conductivity water at 20 ºC using a conductometer (Hanna HI 8733). All the samples were stored in hermetically closed glass bottles at 4 ºC until the volatiles isolation.

### Headspace solid-phase microextraction (HS-SPME)

The isolation of headspace volatiles was performed using a manual SPME fiber with the layer of polydimethylsiloxane/divinylbenzene (PDMS/DVB) obtained from Supelco Co (Bellefonte, PA, USA). The fiber was conditioned prior to use according to the manufacturer instructions. For HS-SPME extraction, honey/saturated water solution (5 mL, 1:1 v/v; saturated with NaCl) was placed in a 15 mL glass vial and hermetically sealed with PTFE/silicone septa. The vial was maintained in a water bath at 60 ºC during equilibration (15 min) and extraction (40 min) and was partially submerged so that the liquid phase of the sample was below the water level. All the experiments were performed under constant stirring (1,000 rpm) with a magnetic stirrer. After sampling, the SPME fiber was withdrawn into the needle, removed from the vial, and inserted into the injector (250 ºC) of the GC and GC-MS for 6 min where the extracted volatiles were thermally desorbed directly to the GC column.

### Ultrasonic solvent extraction (USE)

Ultrasound-assisted solvent extraction (USE) was performed in an ultrasound cleaning bath (Transsonic Typ 310/H, Germany) by the mode of indirect sonication, at the frequency of 35 kHz at 25 ± 3 ºC. Forty grams of *A. fruticosa* honey was dissolved in distilled water (22 mL) in a 100-mL flask. Magnesium sulfate (1.5 g) was added and each sample was extensively vortexed. A mixture of pentane-diethyl ether (1:2, v/v) was used as the extraction solvent. Sonication was maintained for 30 min. After sonication, the organic layer was separated by centrifugation and filtered over anhydrous MgSO_4_. The aqueous layer was returned to the flask and another batch of the same extraction solvent (20 mL) was added and extracted by ultrasound for 30 min. The organic layer was separated in the same way as the previous one and filtered over anhydrous MgSO_4_, and the aqueous layer was sonicated a third time for 30 min with another batch (20 mL) of the extraction solvent. Joined organic extracts were concentrated to 0.2 mL by fractional distillation, and 1 μL was used for GC and GC-MS analyses.

### Gas chromatography and mass spectrometry (GC, GC-MS)

Gas chromatography analyses were performed on a Agilent Technologies (Palo Alto, CA, USA) gas chromatograph model 7890A equipped with flame ionization detector, mass selective detector, model 5975C and capillary column HP-5MS ((5%-phenyl)-methylpolysiloxane Agilent J & W GC column, 30 m, 0.25 mm i.d., coating thickness 0.25 μm). Chromatographic conditions were as follows: helium was carrier gas at 1 mL·min^−1^, injector temperature was 250 ºC, and detector temperature was 300 ºC. HP-5MS column temperature was programmed at 70 ºC isothermal for 2 min, and then increased to 200 ºC at a rate of 3 ºC·min^−1^ and held isothermal for 15 min. The injected volume was 1 μL and the split ratio was 1:50. MS conditions were: ionization voltage 70 eV; ion source temperature 280 ºC; mass scan range: 30–300 mass units. The analyses were carried out in duplicate.

### Data analysis and data evaluation

The individual peaks were identified by comparison of their retention indices (relative to C_9_-C_25_ *n*-alkanes for HP-5MS) to those of authentic samples and literature [27], as well as by comparing their mass spectra with the Wiley 275 MS library (Wiley, New York, USA) and NIST98 (Gaithersburg, MD, USA) mass spectral database. The percentage composition of the samples was computed from the GC peak areas using the normalization method (without correction factors). The component percentages (Table 1, Table 2) were calculated as mean values from duplicate GC and GC-MS analyses. 

### Free radical scavenging activity (DPPH assay)

The free radical scavenging activity was determined by the DPPH assay [28]. Fifty microliters of various concentrations of the extracts (after solvent evaporation) or the honey samples in ethanol were added to 0.004% ethanol solution of DPPH (5 mL). The absorbance measurements commenced immediately at 517 nm using UV–VIS Perkin–Elmer Lambda EZ 201 spectrophotometer and absorbance decrease at room temperature was determined after 1 h for all samples. Pure ethanol was used to zero the spectrophotometer. The absorbance of the DPPH radical without the sample, i.e. the control, was determined. The percent inhibition of the DPPH radical by the samples was calculated in the following way: I% = [(AC(o) – AA(t)) / AC(o)] x 100, where AC(o) is the absorbance of the control at t = 0 min and AA(t) is the absorbance of the sample at t = 1 h. Sample concentration providing 50% inhibition (IC_50_) was calculated from the graph plotting inhibition percentage against sample concentration. DPPH assay was carried out in triplicate for each tested extract and honey.

## Conclusions

HS-SPME and USE followed by GC and GC-MS enabled determination of complementary headspace, volatile and semi-volatile chemical composition for overall characterization of honey aroma (diversity of headspace, volatile and semivolatile compounds). The headspace of the honey was dominated by 2-phenylethanol, while other major compounds were *cis*- and *trans*-linalool oxides, benzaldehyde and benzyl alcohol. 2-Phenylethanol and methyl syringate were the main compounds of ultrasonic solvent extracts, with an array of small percentages of ubiquitous honey VOCs. The extracts exhibited significantly higher free radical scavenging potential than the honey samples at *ca.* 25 times lower concentration ranges (up to 2 g/L) and their scavenging potential is revealed for further more detailed research. These findings indicate the importance of USE extracts, not just for analytical purposes, but also suggest potential as a better indicator of further antioxidant and biological activities of the corresponding honeys. 
